# Supplementary material for: Pathways through homelessness among women in Addis Ababa, Ethiopia: A qualitative study
Source: PLoS One. 2020 Sep 2;15(9):e0238571. doi: 10.1371/journal.pone.0238571 (PMC7467327; doi:10.1371/journal.pone.0238571)
Supplement: Attachment — Submitted filename: Response to reviewers.docx [file pone.0238571.s001.docx]

**Response to reviewers**

Please find herein the point-by-point responses to your comments:

1. ***Sampling***: The word ‘case’ was inserted after ‘typical’ in the sampling strategy subsection as you commented. Check revised manuscript with track changes. About inserting a reference to help justify the method, we think this would not be very vital to this manuscript as our intention is not to teach about sampling methods in qualitative studies. Citations were inserted to provide credit to authorities whose ideas and strong arguments we used to justify our study. Providing a reference as you suggested, we believe, is not relevant here.
2. ***Setting***: In the ‘setting’ sub-section paragraph 2 the statement describing about the shelter being for women and elderly men the following amendment has been made. ‘The shelter was established to provide services to homeless adult women and their dependent children if they had any, and elderly men’. The fact is the shelter took in homeless women together with their children and provided basic services to the children, as well as made it possible to attend school at KG and above.
3. ***Ethics approval***: The unnecessary detailed description about the contents of the participant information sheet was deleted from the subsection ‘ethics approval and consent to participate’ as you suggested. Check revised manuscript with track changes.
4. ***Results***: The following points were addressed:

- The spelling errors in the first paragraph are corrected. ‘on of the participants—‘ was mad ‘one of the ---,‘ ‘---- at least one children—‘ was changed to ‘---at least one child---,‘ ‘step-parent’ was consistently used. Please check manuscript with track changes.
- In the ‘predisposing factors’ sub-section the paragraph which started by the statement ‘some participants had experienced exploitative work conditions---‘ the second statement has been edited to make it better. Please check manuscript with track changes.
- The sentence ‘In some cases participants were opted---‘ has been replaced by ‘In some cases participants opted ---‘ as you commented. Shown by track changes.
- In the paragraph which starts by ‘Some participants had the feeling of shame----‘ the second sentence was changed to ‘They felt it would be an embarrassment to their family, as well as to themselves,----‘ (Shown by track changes)
- The term ‘respondent’ has been replaced by ‘participant’ in several paragraphs of the ‘results’ section. Please check manuscript with track changes.
- Missed words were inserted and grammatical corrections including tense, non-use of contraction and the correct use of plural were made in several parts of the ‘results’ section as shown by track changes.

5. ***Discussion***: The following amendments were made:

- In the first paragraph of the ‘discussion’ section the second sentence was changed into ‘the study was conducted in a country which had cultural norms which gave women a lower and submissive status and child marriage was widely practiced,’ in order to avoid ambiguities.
- The sentence which started by ‘However, the data were presented as were----‘ was removed and replaced by the sentence ‘However, as much participant narrations as possible were provided to give the reader better understanding’. Please check track changes.
